# A Review of the Structure, Preparation, and Application of NLCs, PNPs, and PLNs

**DOI:** 10.3390/nano7060122

**Published:** 2017-05-27

**Authors:** Qianwen Li, Tiange Cai, Yinghong Huang, Xi Xia, Susan P. C. Cole, Yu Cai

**Affiliations:** 1 College of Pharmacy, Jinan University, Guangzhou 510632, China; Elven1608@163.com (Q.L.); xiyiyi213@163.com (X.X.); 2 College of Life Sciences, Liaoning University, Shenyang 110036, China; caitiange@163.com; 3 GuangzhouGuoyu Pharmaceutical Technology Co., Ltd., Guangzhou 510632, China; jodie_615@163.com; 4 Division of Cancer Biology and Genetics, Queen’s University Cancer Research Institute, Kingston, ON K7L 3N6, Canada; 5 Cancer Research Institute of Jinan University, Guangzhou 510632, China

**Keywords:** nanostructured lipid carriers, polymeric nanoparticles, lipid–polymer hybrid nanoparticles, structure, preparation, applications

## Abstract

Nanostructured lipid carriers (NLCs) are modified solid lipid nanoparticles (SLNs) that retain the characteristics of the SLN, improve drug stability and loading capacity, and prevent drug leakage. Polymer nanoparticles (PNPs) are an important component of drug delivery. These nanoparticles can effectively direct drug delivery to specific targets and improve drug stability and controlled drug release. Lipid–polymer nanoparticles (PLNs), a new type of carrier that combines liposomes and polymers, have been employed in recent years. These nanoparticles possess the complementary advantages of PNPs and liposomes. A PLN is composed of a core–shell structure; the polymer core provides a stable structure, and the phospholipid shell offers good biocompatibility. As such, the two components increase the drug encapsulation efficiency rate, facilitate surface modification, and prevent leakage of water-soluble drugs. Hence, we have reviewed the current state of development for the NLCs’, PNPs’, and PLNs’ structures, preparation, and applications over the past five years, to provide the basis for further study on a controlled release drug delivery system.

## 1. Introduction

Nanoparticles are colloidal particles with sizes of approximately 10–1000 nm. These particles may be divided into nanocarriers and nanodrugs. Nanocarriers refer to materials prepared by the dissolution or dispersion of drugs with a variety of nanoparticles, which may be classified as either nanospheres or nanocapsules. The material of the preparation of nanoparticles may be divided into polymers and lipid materials. The former is referred to as PNP, which includes polymer nanocapsules and nanospheres as well as polymeric micelles. The latter is called the lipid nanoparticle, and includes nanoliposomes and NLCs. LPN is a new type of carrier developed in recent years and is a combination of liposomes and polymers. Nanodrugs involve the direct application of micronization and ultrafine powder technologies to the processing of drugs into nanoparticles [1].

NLCs, as a new drug delivery system, appeared in the late 1990s [2]. This delivery system involves the modification of SLNs and a mixture of solid and liquid lipids prepared by heating and cooling crystallization. Compared to SLNs, the advantages of NLCs are as follows: a disordered crystal structure, which can help prevent leakage of the drug load and providing higher drug payload [3]. NLC can be loaded with hydrophobic or hydrophilic drugs with a wide range of drug-loading properties; the carrier material is biodegradable and exhibits low in vivo toxicity [4]. NLCs can also be surface-modified. The carrier exhibits a certain organizational targeting pattern [5]. The physical and chemical properties of the NLC particles are stable.

PNPs are a popular topic in the field of drug delivery and controlled drug release. As a carrier of drug-controlled release delivery, PNPs have the following advantages. PNPs improve the absorption and bioavailability and increase the solubility of drugs; they can cross the blood–brain barrier and allow drug access to the central nervous system. PNPs actively search out and attack cancer cells, thereby improving drug targeting. These carriers penetrate tissue and reside in tissues and cells for a long time to release their ‘cargo’ drugs; they also mainly concentrate in organs, such as the liver and bone marrow, that become enriched in mononuclear macrophages after intravenous therapy, so it can be a therapy for mononuclear phagocyte system (MPS)-related diseases. Compared with liposomes, PNPs can more effectively achieve drug delivery to specific target areas, such as organs or tissues with pathological changes. In this way, they can increase drug efficacy. PNPs can also concurrently reduce the side effects on normal tissues surrounding the target diseased organ. Furthermore, PNPs can enhance drug stability, exhibiting slow and controlled drug release properties, which is particularly relevant to protein drugs. With orally administered drugs, PNPs can act as carriers for proteins, peptides, and nucleic acids, including DNA for gene therapies [6].

The PLN is based on liposome and polymer nanoparticles developed as a new type of drug carrier in recent years. The PLN is composed of a phospholipid shell and polymer core, thereby providing a core–shell structure with the advantages of both the liposome and polymer nanoparticles [7,8]. The advantages of PLNs are many. For example, the solid polymer core serves as a structural framework and provides the hybrid nanoparticle with mechanical stability, shape control, biodegradability, narrow particle-size distribution, and a large surface area [9]. In addition, the lipid shell of the PLN contributes to its high biological compatibility because its properties are similar to that those of the cell membrane, easily combining with a variety of bioactive molecules [10]. The PLN exhibits an enhanced drug loading and encapsulation rate [11]. The lipid bilayer structure is conducive to adsorbing the drug distributed along the bilayer surface and embeds the hydrophobic region of hydrophilic molecules [12,13]. PLNs also load and transport both hydrophilic and hydrophobic drugs [14]. These nanoparticles can adjust the drug release, provide good stability [15], and enable the intracellular targeting of delivered drugs [16]. Particle sizes are <100 nm (similar to virus structure) and can thus allow the application of PLNs as a vaccine adjuvant. As mentioned, the use of PLNs as drug carriers holds great promise for drug development.

NLCs, PNPs, and LPNs have received increased attention in drug discovery and development applications. These carriers can increase the drug distribution to the target organ, change the pharmacokinetic characteristics of drug carriers to enhance the therapeutic effect, and reduce adverse side effects. This review will rapidly survey the state of the art of popular nanoparticles (NLCs, PNPs, and LPNs), including their structure, preparation, and application. Moreover, it will highlight the most important preparation techniques. Finally, it will give a short perspective on the major challenges of drug delivery, which is a potential field of application for LNP, to provide a theoretical background for further research.

## 2. Structural Models of the Drug Carriers

### 2.1. Structural Model of NLCs

NLCs were developed as improvements over SLNs. SLNs are composed of a solid lipid, whereas the NLC lipid phase contains both solid and liquid lipids. NLC increases the amount of liquid lipid such that the nanoparticles form a non-standard shape and stack, resulting in a non-perfect lattice and forming an amorphous structure. As increasing the liquid lipid can enhance the solubility of active substances, and active substances can help with better encapsulation compared to SLNs, NLCs’ lipid material shows more application potential. NLCs augment drug stability and loading capacity, and reduce drug leakage during storage.

**Figure 1:**
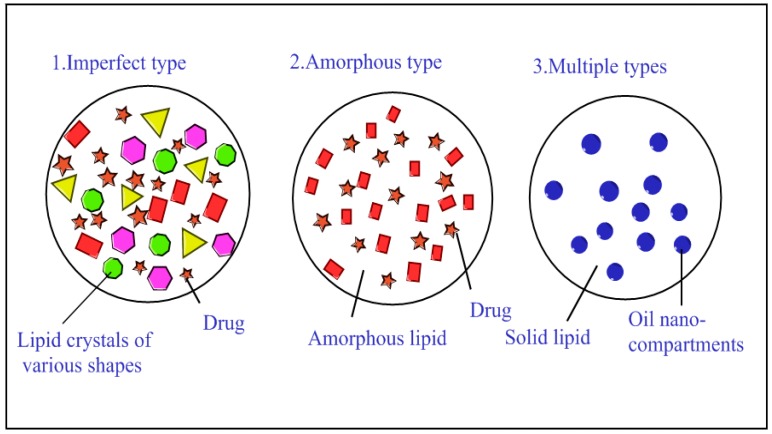
Schematic diagram illustrating structures of NLCs (1, 2, and 3 are disorder structure, amorphous structure and multiple structure, respectively).

Müller et al. [17] examined three kinds of NLC structures: disordered, amorphous, and multiple types (Figure 1). The first kind of structure is the disordered structure, which involves mixed solid and liquid lipids that conform to the disordered state. The disordered lipid structure appears between the crystal and liquid lipid, therefore increasing drug penetration capacity through the lipid layer. The second is the amorphous structure (non-crystalline state). The lack of a crystalline structure can prevent or significantly reduce the leakage of the loaded drug. Since crystals are formed during cooldown, the addition of a mixture of lipids prevents crystal formation. The multiple structure is the third type. This structure contains a higher liquid lipid concentration than the other structures. Drug solubility in liquid lipid is higher than in solid lipid. Therefore, the NLCs can achieve slow drug release and high drug loading, thereby avoiding loss prior to the decomposition of the solid lipid. This structure is similar to that of the water-in-oil-in-water (W/O/W) microemulsions. 

### 2.2. Structural Model of PNPs

**Figure 2:**
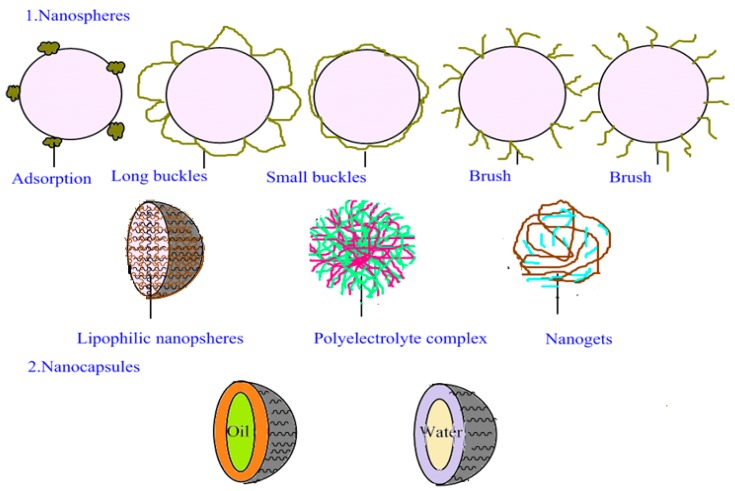
Schematic diagram illustrating structures of two types of PNPs. 1 and 2 are polymer nanospheres and polymer nanocapsules, respectively.

The PNP structure includes polymer nanocapsules and polymer nanocomposites, which are formed by a solid shell surrounding a liquid and semi-solid core at room temperature (15–25 °C) (Figure 2). Lipid-soluble drugs can attain high encapsulation efficiency if the core is a lipid. On the other hand, if the core is water-soluble, water-soluble compounds can be entrapped. The dispersed phase properties of the emulsion and composition of the microemulsion formulation are indices for determining nanocapsule content. Generally, two methods are available for the formation of the liquid core and polymer shell: polymerization occurs between the dispersed phase and the continuous phase [18,19,20,21,22,23,24,25,26], or the precipitation of the surface [27,28,29,30] is formed by emulsion droplets.

Nanospheres are matrix particles, i.e., particles whose entire mass is solid, composed of varying sizes (tens to hundreds of nanometers), and are generally spherical; nanospheres with shape different from a sphere are described in the literature [31,32,33]. Maintaining good nanoparticle dispersion in liquid requires the use of amphiphilic molecules and colloid-protecting agents to provide high stability.

In contrast, nanospheres and nanocapsules serve as drug carriers. Drugs can be enveloped by the nanoparticles or adsorb to their surfaces. Under normal circumstances, molecular fragments from enzymatic degradation are produced in biological media, and these molecular fragments are better stored when the drug is enclosed in nanocarriers. In this case, drug carrier nanocapsules or nanospheres can act as good drug delivery systems.

### 2.3. Structural Model of PLNs

**Figure 3:**
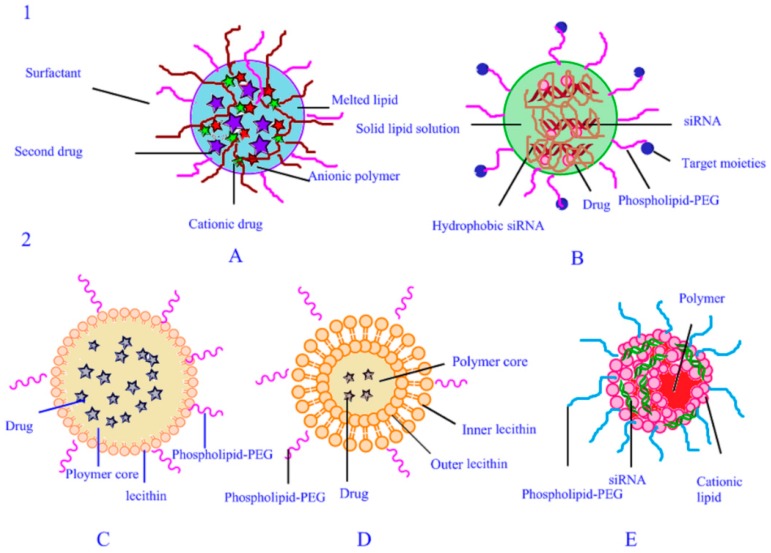
Schematic diagram illustrating the structure of PLNs. 1, monolithic matrix structure; 2, core–shell structure. (**A**) Monolithic matrix PLN with polymer–drug complex; (**B**) monolithic matrix PLN with siRNA–polymer complex; (**C**,**D**) core–shell PLN with drug in the core; (**E**) core–shell PLN with siRNA at the core–shell interface.

Two types of PLN structures exist and are shown in Figure 3. Wu et al. [34,35] examined the structure of the PLNs. The structure of PLNs contains a monolithic matrix PLN and a core–shell PLN. The first type includes a polymer–drug complex and an siRNA–polymer complex, both of which are homogeneously distributed in the solid lipid matrix, while the siRNA–polymer complex is decorated with targeting moieties on the surface. The latter has a core–shell structure, containing two kinds of PLN: PLN with drug in the core and PLN with siRNA at the core–shell interface. Drug in core, is where the polymers and drugs are trapped inside by the solid matrix core, phospholipid, and phospholipid–PEG formed the shell. Preparation for the one-step method produced a phospholipid monolayer. Formed lipid films or liposomes created by the thin-film dispersion method can obtain a phospholipid bilayer. At the core–shell interface of the PLN with siRNA, the core is made of polymer and drug, the shell is formed by mPEG–polymer, and siRNA is entrapped by complexation with the cationic lipid at the interface of the core and the shell.

## 3. Methods for Preparing Drug Carriers

### 3.1. Methods for Preparing NLCs

Many types of protocols are available for the preparation of NLCs. The most commonly used approach is the high-pressure homogenization method, which utilizes both high temperature and high pressure. Another is the low-temperature, high-pressure homogenization method. The techniques for preparing SLNs can also be employed for NLC preparation. These approaches include high-pressure homogenization, ultrasonic emulsion evaporation, solvent dispersion, the film-ultrasonic method, high-temperature emulsion evaporation–low temperature curing, microemulsion, emulsion, supercritical fluid (SCF), membrane contactor, microchannel, and microtube methods. The raw materials, encapsulation efficiencies, and particle sizes in a range of studies on NLC preparation are summarized in Table 1.

#### 3.1.1. High-Pressure Homogenization Method

Methods for preparing NLCs are relatively mature and can be used in mass production and dispersing techniques that do not involve organic solvents. These methods can be divided into high-temperature, high-pressure and low-temperature, high-pressure homogenization protocols. The high-temperature, high-pressure homogenization method is the more commonly adopted and involves the melting of solid lipid materials first before mixing them with liquid lipid and drugs. After mixing, the molten liquid is scattered throughout the aqueous phase, which contains surfactants. The mixture is stirred to form the beginning of an emulsion. Then, by high-speed impact and decompression expansion under an extremely high shear force, fluid droplets are gradually broken into nanoparticles. Generally, high temperatures reduce the viscosity of the mixed liquid, decreasing the particle size but increasing the probability of degrading the drug and the carrier. This method can be successfully used for insoluble drugs and lipophilic ones, but is not entirely suitable for hydrophilic drugs. The advantages are avoidance of organic solvents and large-scale production.

The particle size of the NLC is also related to the homogenization pressure and homogenization times [36]. When the high-pressure homogenization method is used to prepare NLCs [37,38,39,40,41,42,43,44,45,46,47,48,49], the encapsulation efficiency of coenzyme Q_10_ NLC is the highest. El-Salamouni et al. [38] employed a modified high-shear homogenization method to prepare NLCs using glyceryl monostearate (GMS) as the solid lipid, castor oil as the liquid lipid, and Poloxamer188 as the surface active agent. In this procedure, autoclaving at 121 °C for 15 min increased the surface potential and encapsulation efficiency of the drug, thereby generating stable formulations and nanoparticle sizes under 500 nm. Aditya et al. [40] developed and evaluated three lipid nanoparticles, namely, SLN, NLC, and a quercetin nano emulsion, using medium-chain triglyceride (MCT) as the liquid lipid, Imwitor 900 K and soybean phosphatidylcholine as the solid lipid, and Tween 80 and Span 20 as surfactants. The size of the NLCs was measured as 34 ± 6 nm, and the encapsulation efficiency of quercetin was 91%. In this study, the method of preparing the three different lipid nanoparticles was the same, but the physical state and composition of the lipids used differed. These studies showed that different physical states and compositions of the lipids can be used to prepare different lipid nanoparticles. 

#### 3.1.2. Ultrasonic Emulsion Evaporation Method

In this method, the solid lipid, the liquid lipid, and the drug mixture as oil phase are added and dispersed in an aqueous surfactant solution by probe ultrasonication. The sample was then cooled down and solidified to form NLCs. When a stable emulsion is formed, the oil phase is evaporated by heating under reduced pressure, or by evaporation while stirring continuously. Avoidance of heat during the preparation is the most important advantage of this method. Toxicological problems may result from solvent residues from the product obtained by this method.

Ranpise et al. [50] employed GMS as the solid lipid, labrafil and linseed oil as the liquid lipids and lutrol^®^ F68 and polysorbate 80 as the surfactants to entrap lercanidipine hydrochloride. On the other hand, Uprit et al. [51] used soya lecithin as the solid lipid and oleic acid (OA) and tristearin as the liquid lipids to yield a minoxidilan entrapment efficiency of 86.09% and a particle size of 280 nm.

#### 3.1.3. Solvent Dispersion

In the solvent dispersion method, solid lipid, liquid lipid, and the drug are dissolved in a water miscible organic solvent (ethanol, acetone, or isopropanol).Then, the organic solution is slowly added to the water containing the emulsifier, and the NLC is obtained by centrifugation. The drug loading of NLCs prepared by this method generally increases with the mass of the liquid. To further increase the drug loading of NLC, the dispersed phase is usually employed to enclose a saturated drug solution. The advantages of this method are speed, simplicity, and the low requirements of the instrument. The disadvantages of this method are that it is not entirely suitable for industrial production, and there is residual organic solvent.

NLCs are also prepared by the solvent dispersion method [52,53,54,55,56,57]. This method was used by Emami et al. [57]. They were prepared with cholesterol as the solid lipid and OA as the liquid lipid, and poloxamer188 and polysorbate 80 as surfactants, achieving an entrapment efficiency of paclitaxel 72 ± 11.6%.

#### 3.1.4. Film-Ultrasonic Method

In the film-ultrasonic method, the solid lipids, liquid lipids, and drugs are dissolved in an appropriate organic solvent, which is later removed by vacuum evaporation. To form a layer of mixed lipid films, a surfactant aqueous solution is added. Small and uniform NLCs are then produced using an ultrasound probe for ultrasonic dispersion. This method is most often used due to its simplicity and practicality, and its yield of small, uniform particles. However, toxicological problems may result from solvent residues from the product obtained by this method.

In the study of Yang [58] to produce NLCs of curcumin, with CP as the solid lipid, Miglyol 812 as the liquid lipid, and Solutol HS15, and soybean lecithin as surfactant, and curcumin were utilized. Curcumin entrapment efficiency was 96.7 ± 0.146%, and the particle size was 135.3 ± 2.52 nm.

#### 3.1.5. High-Temperature Emulsion Evaporation—Low-Temperature Curing

This method involves independently heating the organic and aqueous phases to the same temperature and then adding the organic phase to the aqueous phase containing an emulsifier so that an emulsion is produced. The volatile organic solvent is then evaporated from the system by heating, and the resulting concentrated liquid is quickly dispersed in ice water (0–4 °C). In this way, a NLC dispersion solution is obtained. The advantages of this method include its simplicity and speed. The disadvantages of this method are that it is not entirely suitable for industrial production, and there is residual organic solvent.

Using a high-temperature emulsion evaporation–low temperature curing method, Yanqiu Li et al. [59] formulated celecoxib NLCs using cetyl alcohol palmitate as the solid lipid, Miglyol 812 as the liquid lipid, and Solutol HS15 and soybean phospholipid as surfactants. The average particle size obtained was 103.5 ± 32.6 nm. Luan et al. [60] employed polyethylene glycol stearate (PEG-SA) and GMS as solid lipids, caprylic/capric triglyceride (CCT) as the liquid lipid, and F68 and soybean lecithin (SL) as the surface-active agents to entrap amoitone B. A NLC particle size of 2.257 ± 1.36 nm and encapsulation efficiency of 68.17 ± 0.94% was obtained.

#### 3.1.6. Microemulsion Method

Using a microemulsion approach, the lipid carrier is heated and melted, and then drugs, emulsifier, auxiliary emulsifier, and deionized water are added to yield a mixture with a transparent appearance and a thermodynamic stability similar to that of oil-in-water (O/W)-type microemulsion. The microemulsion is quickly dispersed in ice water (0–4 °C), forming an NLC dispersion system. The sizes of the nanoparticles and particles from microemulsion and dilution are extremely close to the temperature difference between the cold water and the microemulsion, which is a key factor in preparing small-particle-sized NLCs. Rapid cooling and solidification can prevent the aggregation of some particles. The advantages of this method include its low drug content and simplicity, while the disadvantages are the abundance of auxiliary emulsifier and emulsifier required.

Shao et al. [61] prepared NLC by microemulsion using soya, lecithin GMS as the solid lipid, oleic acid as the liquid lipid, and Tween-80 as the surfactant to entrap paclitaxel. The particle size obtained was 79 nm, and the encapsulation efficiency was 87.1 ± 2.1%.

#### 3.1.7. Melt Emulsification Method

In this protocol, the solid and liquid lipids are heated and mixed. Then the drugs are added to form an organic phase. The organic phase is added to a water phase containing the surfactant and stirred to form a coarse emulsion. High-pressure homogenization is subsequently applied to form the NLCs. This is advantageous because there is no organic solvent residue, no burst release at the initial time, and dispersions with high lipid concentration. The disadvantages of this method are that it is not entirely suitable for industrial production and there is residual organic solvent.

Zhiqiang Tian et al. [62] used this method with Precirol ATO 5 as the solid lipid and Captex 100 as the liquid lipid to entrap fenofibrat, yielding an encapsulation efficiency of 8.5% and particle size of 227.5 nm.

In summary, many types of methods for preparing NLCs are available and are mainly based on the physical and chemical properties of the drugs involved. 

### 3.2. Methods for Preparing PNPs

Two main methods are used to prepare conventional PNPs. The first approach is the direct dispersal of the polymer. For example, polylactide (PLA), polyethylene glycol (PEG), poly(lactic-co-glycolic acid) (PLGA), and ε-hydroxy acid lactone can be re-dispersed in a medium when preparing nanoparticles [63]. The second approach is the direct preparation of nanoparticles by the monomer polymerization process. PNPs can be prepared by the emulsion evaporation method, the double emulsion evaporation method, the dialysis method, the improved thin-film dispersion method, nanodeposition, supercritical fluid technology, the ionic gelation method, and the self-assembly method. Various methods for the preparation of PNPs are summarized in Table 2.

#### 3.2.1. Emulsion Evaporation Method

This method involves the dissolution of a polymer in methylene chloride, chloroform, ethyl acetate, or another organic solvent. The drug is dissolved or dispersed in the polymer solution to form an organic phase. The organic phase is then uniformly and slowly added to the water phase and emulsified to form an O/W system. The emulsifier or other surface active agents used in this system include gelatin and polyvinyl alcohol (PVA), Span 80, and Poloxamer188. After a stable emulsion is formed, the organic solvent is evaporated by heating under reduced pressure. Avoidance of heat during the preparation is the most important advantage of this method. Toxicological problems may result from solvent residues from the product obtained by this method.

Hoa et al. [64] adopted the emulsion evaporation method to prepare PNP; Eudragit E100 and Eudragit RS were used as the polymer to entrap ketoprofen and particle sizes of 50–150 nm.

#### 3.2.2. Double-Emulsion Evaporation Method 

The method involves water-soluble drugs as the internal phase dispersed throughout an organic phase containing PLGA or other carriers to form a W/O as the start of the emulsion. The start of the emulsion is then dispersed in an external aqueous phase to form a W/O/W type double emulsion, removing the organic solvent during preparation. W/O/W double emulsion preparation technology can be applied to preparing water-soluble drug nanoparticles. Toxicological problems may result from solvent residues from the product obtained by this method.

Zhang et al. [65] and Sheng et al. [66] prepared PNPs by the double-emulsion evaporation method. PLGA and PLA were used as polymers, and the sizes of the nanoparticles were 188 and 100–200 nm, respectively. 

#### 3.2.3. Dialysis Method

In the dialysis method, the polymer and drug are dissolved in an organic solvent, and then the mixed solution is dialyzed. Because of their large sizes, the PNPs remain frapped by the dialysis membrane. The drug concentration in the external medium is measured over time until it stabilizes. This and the free drug concentrations on the two sides of the dialysis membrane have equilibrated. This particular point in time is termed as the free-drug dialysis balance time. The concentration of the drug in the medium is then calculated and regarded as the free drug concentration, which is used, in turn, to calculate the drug encapsulation efficiency. This method is simple and avoids the use of surfactants. However, the wide particle size distribution is a disadvantage, and toxicological problems may result from the solvent residues obtained by this method.

Liu et al. [67], Zhang et al. [68], and Liang et al. [69] used C (KRG-Dy) (LHRyK), dimethylaminoethyl methacrylate (DMAEMA) and rdroxyethl acrylate(HEA), and poly(ethyleneimine) (PEI), respectively, to load paclitaxel, folic acid (FA), and 4-bromine-1,8-naphthalene anhydride. The particle sizes were 131.7 ± 2.3 nm, 275 nm, and 5–10 nm, respectively.

#### 3.2.4. Improved Thin-Film Dispersion Method

Using this method, the drug is dissolved in an organic solvent and subjected to vacuum evaporation, causing the drug to form a film on the vessel wall. The polymer is then dissolved in an organic solvent, and polymer is completely dissolved in the film. The solvent is removed under reduced pressure, such that the drug and the carrier are evenly mixed into a film. After the addition of the aqueous phase, the ultrasonic and filtration membrane was obtained. This method is most often used due to its simplicity and practicality, and its ability to yield small and uniform particles. However, toxicological problems may result from the solvent residues obtained by this method.

Yang et al. [70] used this method with mPEG-LPEI-photo as the polymer, to entrap 10-hydroxycamptothec (HCPT), achieving an encapsulation efficiency of 92.6 ± 1.1% and a particle size of 155 ± 9.6 nm.

#### 3.2.5. Nanoprecipitation Method

In this approach, the polymer and drug are dissolved in an organic solvent to form an organic phase. The organic phase is then added slowly to the aqueous phase to form an emulsion and then the organic solvents are removed. The basic principle of this method is to deposit a polymer from a lipophilic solution, which is a mixture of polar organic solvent and water. The nanoprecipitation method is a rapid and reproducible method for preparing PLNs. However, toxicological problems may result from the solvent residues obtained by this method.

Deng et al. [71] employed this method with PLGA as polymer, and folic acid conjugated chitosan oligosaccharide (F-CS) as surface modification to entrap paclitaxel and obtain a particle size of 321 ± 0.76 nm.

Liu et al. [72] prepared PNPs by the nanoprecipitation method using PEG–PLGA Me as polymer to entrap DNase I and form a particle size of 89.7 nm.

#### 3.2.6. Supercritical Fluid Technology

Use of supercritical fluid technology has become a popular method for preparing PNPs. This approach employs an environmentally friendly solvent and hence allows the production of high-purity particles without organic solvent residue. There is now a considerable body of literature reporting on the preparation of drug carrier particles using this technique. This method includes rapid expansion of supercritical solution method, (RESS) and supercritical anti-solvent method, (SAS).

For the RESS method, polymer and drugs are dissolved in supercritical fluid at a certain temperature and pressure, and then the supercritical solution is ejected from a nozzle with rapid pressure expansion so that we can see a mechanical disturbance. The rapid expansion of the supercritical fluid can form an extremely high degree of supersaturation; the solute forms a crystal nucleus in an instant, and the growth of the crystal nucleus is completed in a relatively short period of time, leading to a condensation of particles or subparticles. 

In the SAS method, the drug is dissolved in an appropriate organic solvent. Under high pressure, a sufficient amount of supercritical fluid is selected as an anti-solvent. Given the organic solvent solubility in supercritical fluid, the ability to dissolve the polymer and drug dramatically decreases. Supercritical fluids are highly soluble in organic solutions, and then the volume of the solution is expanded and the cohesion is reduced, thereby reducing the solubility of the polymer and the drug. Thus, the supercritical fluid dissolves the organic solution, the organic solution rapidly becomes supersaturated, and the polymer and the drug precipitate to form minute particles.

Zhao et al. [73] prepared bovine insulin nanoparticles by the supercritical fluid technology method with different proportions of PEG and PLA copolymer as carriers. The average particle size was 400–600 nm. More than 90% of bovine insulin and PEG were trapped in the PLA nanoparticles.

### 3.3. Methods for Preparing PLNs

The various methods used for preparing PLNs are summarized in Table 3.

#### 3.3.1. Two-Step Method

The two steps of this method refer to independent processes for preparing the polymer core and the lipid shell. The structure of the desired lipid shell polymer core is obtained by hydration, ultrasound, or extrusion. Two forms of mixing lipid membrane and polymer nanoparticles are also adopted. The lipid membranes and polymer nanoparticles are directly hydrated or are added to preformed lipid membranes. Lipid membranes are formed on the surface of polymer nanoparticles by electrostatic interactions [74]. This process usually requires a low-energy mixing process, and the mixture is heated to a temperature above the lipid phase transition temperature (Tm) to restructure the lipid and polymer nanoparticles. The phospholipid phase transition temperature is the temperature at which the acyl chain of phospholipids is converted from crystalline to liquid. At the phase transition temperature, the activity of the acyl chain is enhanced and the permeability of the liposome membrane is improved. High melting point lipids are included with Tm above 37 °C, such as phosphatidyl choline (dipalmitoyl phosphatidylcholine, distearoyl phosphatidyl choline, 1-myristoyl-2-stearoyl-*sn*-glycero-3-phosphocholine, 1-palmitoyl-2-stearoyl-*sn*-glycero-3-phosphocholine and 1-stearoyl-2-palmitoyl-*sn*-glycero-3-phosphatidylcholine), phosphatidylglycero (1,2-Distearoyl-*sn*-glycero-3-phosphoglycerol-Na and 1,2-Dipalmitoyl-*sn*-glycero-3-phospho-(1′-*rac*-glycerol)-Na), phosphatidyl ethanolamine (dimethyl-2-(dimethylphosphino)ethylphosphine, distearoyl phosphoethanolamine and dipalmitoylphosphatidylethanoiamine), phosphatidylserine (distearoyl phosphatidylserine-Na, dipalmitoyl phosphatidylserine-Na) and phosphatidic acid (2,2-dimethylol propionic acid-Na, distearoyl propionic acid-Na, and dipalmitoyl phosphate-Na) [75]. Spray-drying and soft lithography particle molding have been employed to prepare LPNs in a new manner. PLNs are then obtained by centrifugation of the non-adsorbed lipid membranes, micelles, and free polymer nanoparticles. However, during the two-step method, the encapsulation efficiency of the water-soluble drug may decrease, and the drug may leak from the polymer core before the phospholipid shell is formed [76]. Therefore, this method is limited by its complex technology and low efficiency.

#### 3.3.2. Double-Emulsion Solvent Evaporation Method

The double-emulsion solvent evaporation method is also a two-step method for preparing PLNs. It is a better choice for some substances, such as water-soluble drugs, that are insoluble in any organic solvents and cannot be dissolved together with the polymer. For such substances, it is dissolved in the aqueous phase and emulsified in an oil phase containing the polymer and the lipid to form a W/O emulsion. The emulsion is emulsified in an aqueous phase containing the lipid–PEG to form W/O/W emulsion, and then the organic solvent is removed by reduced pressure and evaporation, giving rise to the LPNs. Avoidance of heat during the preparation is the most important advantage of this method. Toxicological problems may result from solvent residues, however.

Zhao et al. [77] used this method with methoxy polyethylene-poly(lactic-*co*-glycolic acid) (MePEG-PLGA) as polymer, Arg-Gly-Asp (RGD) peptide-polyethylene glycol (PEG)–cholesterol (Chol) copolymers(PEG-RGD-Chol), and lecithin/cholesterol as lipid to entrap curcumin. The entrapment efficiency of the nanoparticles was 96 ± 0.6%, and the particle size was 216.6 ± 4.7 nm.

Zhao et al. [78] also utilized this method with mPEG–PLGA as polymer, lecithin, 1,2-distearoyl-*sn*-glycero-3-phosphoethanolamine-*N*-[methoxy(polyethylene-glycol)-2000 (DSPE–PEG2000), and cholesterol as lipid to entrap gemcitabine and Hypoxia-inducible factor 1a (HIF1a) siRNA together. The particle size was 141.8 nm. Finally, Li et al. [79] employed mPEG-PLA as a polymer and phospholipid as the lipid to encapsulate 5-fluorouracil. The encapsulation efficiency of the nanoparticles was 22.6%. When cholesterol was used as the lipid to entrap Oxaplatin, the encapsulation efficiency of the nanoparticles was 26%. When DSPE–PEG2000 and 1,2-distearoyl-*sn*-glycero-3-phosphoethanolamine-*N*-[maleimide(polyethylene-glycol)-3400] (DSPE–PEG3400-Mal) were used as lipids to enclose camptothecin, the encapsulation efficiency of the nanoparticles was 96%.

#### 3.3.3. One-Step Methods

One-step methods of PLN preparation include the modified solvent extraction/evaporation and nanoprecipitation method. Compared with the two-step methods, one-step methods save time and are convenient, so have been studied to a greater extent and used in more applications. Examples of one-step methods are the emulsion evaporation method, the modified solvent extraction/evaporation method, the ultrasonic method, the high-pressure homogenization method, thin-film hydration, the ultrasonic dispersion method, and the nanoprecipitation method.

#### 3.3.4. Emulsion Evaporation Method

This method is used for substances that are soluble in a water-immiscible solvent (i.e., oil phase). In this approach, a polymer is dissolved in dichloromethane chloroform, ethyl acetate, or another organic solvent. Meanwhile, the drug is dissolved or dispersed in the polymer solution to form an organic phase. The lipid is dissolved in water, and the organic phase is then added to the aqueous phase to form an O/W system. When a stable emulsion is formed, the organic solvent is evaporated by heating under reduced pressure, or by evaporation while stirring continuously. Avoidance of heat during preparation is the most important advantage of this method. Toxicological problems may result from solvent residues, however.

Using this one-step method, Cai et al. [80] used Pylori adhesion material pectin sulfate (PECS) as the polymer and rhamnolipid as the lipid to encapsulate amoxicillin; the particle size was 200 nm. In addition, Li et al. [81] employed curcumin–PLGA as the polymer, and 1,2-dipalmitoyl-*sn*-glycero-3-phosphocholine (DPPC) and DSPE-PEG as the lipids to entrap human fibronectin, and the particle size was ~150 nm.

#### 3.3.5. Modified Solvent Extraction/Evaporation Method

The modified solvent extraction/evaporation method [89] was reported for the first time in a study that encapsulated paclitaxel [90,91]. The polymer and the drug were dissolved in a water-insoluble organic solvent such as dichloromethane, chloroform, or ethyl acetate. The lipid was then mixed with water to form an aqueous phase. The organic phase was added to the aqueous phase, the suspension was dispersed by ultrasound, and the organic phase was transformed into tiny nanoparticles. The nanoparticles were coated with a lipid layer, and the samples were separated and purified by organic solvent removal. The suspensions obtained are fairly dilute due to the limited solubility of the lipids in the organic solvents used. The concentration of PLNs using this method is lower. Additionally, toxicological problems may result from solvent residues by this method.

Colombo et al. [82] used this method with PLGA as the polymer and phospholipids and dioleoyltrimethyl-ammoniumpropane (DOTAP) as the lipids to entrap siRNA. The encapsulation efficiency of the nanoparticles was 63.3 ± 5%, and the particle size was 213–286 nm.

#### 3.3.6. Ultrasonic Method

In this approach, the melted lipid, polymer, and drug are added and dispersed in an aqueous surfactant solution under probe ultrasonication. The sample was then cooled down and solidified to form NLCs. This method is easy and requires ordinary tools that are available in a laboratory. The disadvantage of this method is the low dispersion quality. The dispersion quality of the PLNs produced by these methods is often affected by the presence of microparticles, leading to physical instability upon storage. The lipid concentration is low (<1%) and the surfactant concentration is comparatively high. Metal contamination is the other important problem with ultrasonication. 

Mandal et al. [83] used this method with polycaprolactone (PCL) as the polymer and hydrogenated soy phosphatidylcholine (HSPC) and DSPE-PEG2000 as the lipids to entrap erlotinib. The entrapment efficiency of the nanoparticles was 66%, and the particle size was 170 nm.

#### 3.3.7. High-Pressure Homogenization Method

In this method, the polymer and drug are heated and melted, and then dispersed throughout the aqueous phase containing the lipid by mixing to obtain a start of the emulsion. The start of the emulsion is then subjected to high pressure, high-speed impact, and decompression expansion, after which the fluid droplets are gradually broken down by high shear forces to the desired nanoparticle diameter range. This method can be successfully used to insoluble drugs and lipophilic, but is not entirely suitable for hydrophilic drugs. The advantages are avoidance of organic solvents and large-scale production.

Ramasamy et al. [84] employed this method with chitosan (CT) or anionic (HA) as the polymer, and Compritol 888 soybean and lecithin ATO as the lipids to encapsulate dextran doxorubicin. The particle size was approximately 265 nm.

Seedat, N. et al. [85] used eudragit RS100 (EUD) as polymer, and four different lipids glyceryl tripalmitate (GTP), OA, CT, and sodium alginate (ALG), to entrap vancomycin (VCM). The encapsulation efficiencies of the nanoparticles were 27.8%, 41.5%, 54.3%, and 69.3%, respectively.

#### 3.3.8. Thin-Film Hydration and Ultrasonic Dispersion

In this method, the polymers and drugs are dissolved in an appropriate organic solvent, subjected to vacuum evaporation to remove the organic solvent, and used to produce a layer of polymer film. A lipid aqueous solution is added, and the mixture is subjected to ultrasonic dispersion using an ultrasound probe to form PLNs. This method is most often used due to its simplicity and practicality, and its ability to yield small and uniform particles. However, toxicological problems may result from solvent residues.

Using this method, Zhang et al. [86] utilized poly(ε-caprolactone)-poly(ethylene glycol)-poly(ε-caprolactone) (PCL–PEG–PCL) as the polymer, and DSPE-PEG2000 as the lipid to entrap paclitaxel. The particle size was 279.9 ± 8.7 nm.

#### 3.3.9. Nanoprecipitation Method

The nanoprecipitation method is a rapid and reproducible method for preparing PLNs. In this approach, polymers and drugs are first dispersed in a solvent miscible with water (e.g., acetone and acetonitrile). Then, the solution is dropped into a lipid-containing aqueous phase, and the nanoparticles are mixed by spinning and homogenization. This preparation method is based on the emulsification method, with the use of a lipid substitute for surface-active agents. Lipids, polymers, and drugs are dissolved in the oil phase, and the oil phase is mixed with the water phase to form an O/W emulsion. The hydrophobic region of the lipid attaches to the polymer core, and the hydrophilic end of the lipid extends to the aqueous phase to effectively form a PLN. Toxicological problems may result from solvent residues from the product obtained by this method.

Using this method, Archer et al. [87] employed PCL as the polymer and phospholipid as the lipid to encapsulate methotrexate. The methotrexate entrapment efficiency of the nanoparticles was 80–90%, and the particle size was 150–300 nm.

In addition, Zhao et al. [88] used PLGA as the polymer, mPEG-S-S-C16, DSPE-PEG2k folate, and lecithin as lipid to entrap docetaxel. The entrapment efficiency of the nanoparticles was 82 ± 2%, and the particle size was 100–120 nm.

## 4. Applications of Nanostructured Lipid Carriers

### 4.1. Applications of NLCs

NLCs can be used in a wide variety of drug delivery systems such as oral drug delivery system, transdermal drug delivery system, injection drug delivery system, and gene transfection. 

Zhuang and Chen [92,93] studied NLC as a carrier for oral administration. They prepared insoluble drugs or ones that were easily damaged by digestive enzymes such as antibiotics and enzymes for oral administration. The particles can be absorbed through the lymphatic system. The controlled-released particles can slow down drug degradation and elimination, thereby enhancing the bioavailability of drugs. 

Puglia and Junyaprasert [94,95] investigated a carrier for transdermal delivery systems. NLC was similar to SLN: its small particle size can form a thin film on the skin, so that drugs entrapped in NLC avoid chemical decomposition. Meanwhile, NCL controls drug release, protects the skin, and prevents skin atrophy from repeatedly taking the medication. 

Joshi [96] researched the injection drug delivery system and showed that NLC made into a colloidal solution or freeze-dried powder can be used for intravenous injection to achieve sustained release and prolong the residence time of the drug in the circulatory system or target site. 

Zhang [97] revealed that NLC could be used as a transfer gene carrier, generally divided into two categories: viral vectors and non-viral vectors. The viral vector can efficiently transport genes, but the carrier immunogenicity, tumorigenicity, limited amount of transport DNA, and higher costs restrict its application. However, the NLC is a non-viral vector, so it cannot only overcome the above shortcomings of viral vectors, but also can be sterilized and freeze-dried as a result of the high stability.

### 4.2. Applications of PNPs

PNPs, as a carrier system, hold broad prospects for medical application depending on the characteristics of the particular PNPs. These prospects include antitumor drugs, anti-infective drugs, polypeptide drugs, transdermal medications, and reagents used for diagnostic purposes.

#### 4.2.1. Carriers for Antitumor Drugs

The PNP drug delivery system is a highly valuable application for antitumor therapy. PNPs can control drug release, prolong drug retention time in the body, and improve drug targeting to the tumor site (and thus efficacy) while reducing adverse side effects. Furthermore, the increased permeability of PNP enhances the penetration of the drug-loaded nanoparticles in tumor cells. The mechanism of PNP transport across the cell membrane differs from that of the free drug. PNPs are incorporated by endocytosis and thus their uptake is not affected by the drug transporters in the cell membrane. Thus a combination of antitumor drugs and PNPs can circumvent the multi-drug resistance of tumor cells mediated by drug transporters.

Abraham et al. [98] studied block co-polymer nanoparticles with a degradable cross-linked core and low-molecular-weight PEG corona for anti-tumor drug delivery. Doxorubicin, as a model anti-tumor drug, was loaded into the nanoparticles. Results obtained using flow cytometry showed that doxorubicin-loaded cross-linked particles were taken up by SH-EP cells in quantities comparable with free doxorubicin. These results support the crosslinking of the nanoparticles as carriers for antitumor drugs.

Miao et al. [99] explored anti-tumor drugs using amphiphilic hyperbranched copolymer particles to entrap 2-benzoylpyridine 4-ethyl-3-thiosemicarbazone (Bp4eT). Encapsulation in NPs was done in an effort to increase the anti-tumor activity of this agent by facilitating its delivery to tumor cells. PNPs can control the drug release profile with increased release at acidic pH. Anti-tumor proliferation assays showed that both free drug and drug-encapsulated NPs markedly inhibited tumor cell proliferation in a time- and concentration-dependent manner. Direct microscopic observations revealed that the fluorescent NPs were taken up by cells and localized, in part, in organelles consistent with lysosomes. Amphiphilic, hyperbranched HPAECO and PLA can be used as effective carriers for the intracellular delivery of antitumor agents.

#### 4.2.2. Carriers for Antibiotic Drugs

In many infected bacterial cells, penicillin, cephalosporin, and aminoglycoside activity can be limited. The decrease in intracellular activity concentrations (and activity) of these antibiotics may be partly explained by differences in cell permeability. PNPs can improve targeting by using loading antibiotics in a polymer carrier. If the surface of the polymer nanoparticles is chemically modified, the PNPs can circulate in the bloodstream for longer periods, ultimately increasing PNPs availability to combat infection. As such, PNPs can enhance the effects of antimicrobial drugs.

Ungaro et al. [100] investigated the pulmonary delivery of tobramycin (Tb) using a PLGA nanoparticle powder. They evaluated drug entrapment efficiency, the lung deposition pattern of drug release rate, and hydrophilic polymer properties. In particular, the group studied the design and development of a pulmonary delivery system for the antibiotic tobramycin. In vivo distribution studies showed that PVA-modified alginate/PLGA nanoparticles reached deep into the lung, whereas chitosan-modified nanoparticles were found in the respiratory tract and on the surface of the lung epithelial lining.

#### 4.2.3. Carriers for Skin Protein Drugs

With the advancements in biotechnology in recent years, the research and development of peptide and protein drugs for the prevention and treatment of disease have become a hot topic in pharmaceutical science. However, peptide/protein drugs are easily degraded by gastrointestinal proteases and these drugs are typically available in high-molecular-weight forms with low oral absorption and substantially restricted clinical applications. The biological half-life of such drugs is generally short, and frequent drug administration is required, resulting in psychological and physical inconvenience for patients. Preparing nanoparticles that can protect peptide drugs from protease degradation can improve drug stability in the gastrointestinal tract and improve drug uptake and transport by intestinal mucous membranes. 

Teekamp et al. [101] studied the different production techniques for microparticles and nanoparticles suitable for peptide and protein delivery. The ability of PNPs to improve the stability of the biological macromolecules related to this production technology was evaluated by comparing different production technologies suitable for peptide and protein release.

#### 4.2.4. Carriers for Transdermal Drug Delivery

PNPs have the advantages of high drug entrapment efficiency, good controlled drug release properties, and protection of labile drugs [102]. The large specific surface area of the nanoparticles can make them accumulate on the surface of the skin. Smaller particle sizes can also facilitate a higher accumulation on the surface of the skin, which is beneficial for the transdermal permeation of drugs [103].

Zhan et al. [104] reviewed the progress of the application of PNPs in transdermal drug delivery. Transdermal drug delivery systems (TDDS) have attracted more and more attention due to their potential to avoid hepatic first-pass metabolism, maintain a constant and prolonged drug level, and provide a local target and convenient administration. However, the stratum corneum (i.e., the outermost layer of the skin) acts as a biological barrier, which limits the transdermal delivery of drug molecules, especially hydrophilic ones. For this reason, the development of effective methods to enhance the penetration of drugs through the skin has become important. In recent years, as a kind of drug carrier for TDDS, PNPs have become popular because of their excellent properties such as high entrapment efficiency, reduced enzymatic degradation, controlled drug release rate, and large specific surface area such that they can easily accumulate on the surface of the skin to promote the penetration of the drug.

#### 4.2.5. Applications in Diagnostic Reagents

PNPs can be used to enhance the use of diagnostic reagents in many ways. The detection of cancer cells can be improved by using PNPs. The PNPs can serve as a carrier for negative contrast agents in vivo and used in diagnostic radiology, such as in computed tomography and magnetic resonance imaging. The in vivo use of PNPs with diagnostic reagents is enabled by the particles’ good distribution characteristics in vivo.

Jeon et al. [105] studied the diagnosis of malaria on the basis of cationic polymers and gold nanoparticles. The proposed method was based on the interaction among the Plasmodium lactate dehydrogenase (pLDH), (a biomarker for malaria), the pL1 aptamer against Plasmodium vivax lactate dehydrogenase (PvLDH) and Plasmodium falciparum lactate dehydrogenase (PfLDH). In addition, the cationic polymers, poly(diallyldimethylammonium chloride) (PDDA) and poly(allylamine hydrochloride) (PAH), aggregate gold nanoparticles (AuNPs) that change in color from red to blue depending on the concentration of pLDH. This sensor can successfully detect the pLDH protein and consequently diagnose malaria.

### 4.3. Applications of PLNs

PLNs, as a new carrier system in the medical field, can be used for a wide range of applications, including drug delivery, gene delivery, and diagnostic imaging.

#### 4.3.1. Drug Delivery

PLNs are used in drug delivery, with anti-tumor drug delivery being the most common application. As carriers in drug delivery, PLNs are suitable for intracellular drug release, targeted drug delivery, enhanced cell killing, protection of normal tissue, and reduction of adverse effects to the system [106].

Sengupta et al. [107] and Ebos et al. [108] have employed PLNs as carriers for combretastatin (an anti-angiogenesis drug) and doxorubicin (anticancer, antibiotic). The former drug inhibits the growth of tumor cells by blocking the blood supply to the tumor cells, whereas doxorubicin directly kills existing tumor cells by interacting with topoisomerase II and inducing apoptosis.

#### 4.3.2. Gene Delivery

PLNs are used to delivery genes that can be transferred by non-viral vectors, such as cationic liposomes (DOTAP) and cationic polymers (PEI). Although liposomes and polymers have been shown to be effective both in vitro and in vivo, their action is limited by instability after administration. Moreover, the cationic surface of some polymers easily combines with serum proteins, leading to rapid removal from the systemic circulation and non-specific uptake by non-target tissues. Therefore, liposomes and polymers are not optimal gene delivery vehicles [109]. 

Li et al. [110] found that PLNs exhibit high stability and good biocompatibility compared with liposomes and PNPs. In one preparation of LPNs containing DNA, unmodified LPNs were composed of a PEI core and a polycarbonate three OA glycerin ester two stearic acylphosphatidylcholine lipid shell. Modified LPNs were delivered to HEK293 cells and MB MDA 231 breast cancer cells. The transfection efficiency was higher than when using liposomes; the colloid was more stable, and the toxicity to the HEK293 and MB MDA 231 breast cancer cells by the PLNs was low.

#### 4.3.3. Delivery of Diagnostic Imaging Agents

PLNs are also employed in the delivery of diagnostic imaging agents. In recent years, the high stability and biocompatibility of PLNs in such an application have been studied.

Mieszawska [111] studied AuNPs as biological imaging contrast agents (quantum dots and inorganic nanocrystals). Through the nanoprecipitation method, AuNPs and quantum-dot-loaded PLNs can be prepared. The quantum dots located in the center of the lipid and polymer, and then linked by an esterification reaction, thus forming polymer PLGA conjugates. In vitro experiments successfully showed the bio-imaging of mouse macrophages by PLNs of AuNPs and quantum dots.

## 5. Conclusions

Nanoparticles are rapidly developing as drug carriers. Lipid nanoparticles are widely employed in drug delivery. Many different types of lipid nanoparticles have been engineered, such as lipid nanocapsules, lipid drug conjugates, SLNs, and NLCs. NLCs involve the modification of SLNs. The structural arrangement of solid and liquid lipids of NLCs is not fixed in design. Thus, the structure is flexible, and high encapsulation efficiency and drug loading can be attained, and leakage impeded while retaining the features of SLNs. NLCs, as a new generation of lipid nanoparticles, offer many advantages and a wide range of applications, such as in drug delivery, with broad prospects for further development. NLCs are the most important type of drug delivery and are prepared mainly using HPH, which is a well-established technique; however, the disadvantages related to this production method (high operating temperatures and cavitation forces), as well as the need for encapsulating many types of drugs with different physico-chemical features and various stability and solubility problems, led to the development of new types of lipid nanoparticles and innovative preparation methods. At present, research on NLCs is limited to preclinical studies, with clinical applications remaining far from realization. There are some limitations, such as the presence of organic solvent residue, uneven distribution, complex production process, poor stability, and so on. 

Therefore, PNPs entered the field as a better drug carrier. Compared to NLCs, PNPs not only can effectively transport the drug to a specific target site, such as the organs or tissues affected by the disease, but also increase the efficacy of drugs in diseased tissue. Meanwhile, PNPs can reduce the side effects on the surrounding normal tissue and organs, enhance the stability of the drug, and make the release more gradual and better controlled. Many have benefitted from the ability to control or adjust the drug release, which is achieved through the modification of nanoparticle surfaces, development of new carrier materials, and the study of targeted drugs. Currently PNPs mainly dissolved the drug after coating or adsorption on the nanoparticles’ matrix, which can be used to prepare nanospheres and nanocapsules. Nanospheres are a homogeneous system; drugs can evenly disperse therein. Nanocapsules are a porous system, which allows drugs entrapped to pass through a single polymer membrane. With improvements in the preparation methods for nanoparticles, the development of new materials, and further study on the mechanisms of drug uptake and distribution in vivo, polymer nanoparticles are expected to enter the market for the benefit of mankind in the near future. How to achieve more reliable nanocarriers, more sensitive and accurate drug targeting, more effective treatment, more convenient protocols, better encapsulation rates and release times, and higher stability than the present technology are key objectives of ongoing research.

PLNs are a new kind of drug carrier that have the advantages of both liposome and polymer carriers. PLNs can be adopted not only for single-drug delivery but also as carriers of combinations of drugs. A large amount of biocompatible polymers and lipids are available to be selected, which aids the biocompatibility to surface modification and prevents the leakage of water-soluble drugs, as well as enhancing the preparation method. At present, existing PLN research focuses on their preparation and in vitro studies and targeted research. The stability of new drug products is an essential prerequisite. Therefore, the long-term physical and chemical stability of these hybrid nanoparticles in various environmental stress conditions needs to be systematically evaluated. In important challenging research areas such as optimizing target ligand density, reducing adverse reactions, in vivo studies, and large-scale production are necessary for achieving major advancements in this field.

## Figures and Tables

**Table 1:** Summary of methods used to prepare NLCs.

Drug	Solid Lipid	Liquid Lipid	Surfactant	Method	Entrapment Efficiency/Particle Size	Ref.
CoenzymeQ10	Hard Stearin	GTCC	Alkyl polyglycoside	HPH	99.58 ± 0.0061%	[37]
Brimonidine base	GMS	Castor oil	Poloxamer188	HPH	10.51%	[38]
Lornoxicame	Compritol888ATO, Lanette O	OA	Pluronic F68	HPH	97.89 ± 0.25%	[49]
Quercetin	Imwitor 900 K	MCT	Tween80, Span20, Soybean lecithin	HPH	91%	[40]
Saquinavir mesylate	Precirol ATO5	Miglyol812	Tween80, Poloxamer188	HPH	--	[41]
UvinulT 150	ACETEM	Odograph, Hydrogenated palm	OlivemR800 OlivemR1000	HPH	314 ± 4 nm	[42]
thymoquinone	Lipoid S100	oil	Sorbitol, Thimerosal, Polysorbate80	HPH	75 ± 2.4 nm	[43]
Docetaxel	Stearic acid, Glycerin monostearate	Olive oil, MCT, OA	Pluronic F68, Cremophor EL	HPH	60.5 ± 5.0%	[44]
β-carotene	Hydrogenated palm kernel	Isopropyl palmitate	Sorbitan monopalmitate, Polysorbate80	HPH	259 ± 4.24 nm	[45]
Tocolsenzophenone-3	Glycerides, Carnauba wax	Isodecyloleate	Poloxamer188, Polysorbate80	HPH	91%	[46]
β-Elemene	GMS	Maisine35-1, Labrafil	Polysorbate80, soybean lecithin	HPH	138.9 nm	[47]
					82.11%	
Fenofibrate	Compritol888, ATO	M1944CS, Labrafil	Soya lecithin, Polysorbate80	HPH	99%	[48]
					84.9 ± 4.9 nm	
4-dedimethylamino sancycline	Stearic acid, Glycerin monostearate	OA. MCT	LutrolF68	HPH	90–96%	[49]
					<200 nm	
Lercanidipine HCl	GMS	Linseed oil, Labrafil	Polysorbate80	Ultrasonication and emulsion evaporation	--	[50]
Minoxidil	Soya lecithin	OA	Polysorbate80	Ultrasonication and emulsion evaporation	86.09%	[51]
					280 nm	
Dexamethasone	glycerol trilaurate	Tristearin, Chain Triglycerides Miglyol812	Phospholipids	Solvent diffusion	86.7 ± 3.9%	[52]
Isoliquiritigenin	Soya lecithin, Cholesterol	Glycerol	Polysorbate80, Poloxamer188	Solvent diffusion	96.74 ± 1.81%	[53]
					160.73 ± 6.08 nm	
Lovastatin	Cholesteryl oleate, cholesterol	Trioleate	Soybean lecithin	Solvent diffusion	96.2 ± 1.3%	[54]
					13.8 ± 2.2 nm	
Celastrol	Precirol ATO-5	Labrasol	Lecithin, TPGS, Poloxamer188	Solvent diffusion	88.6 ± 0.37%	[55]
					132.3 ± 25 nm	
Gentiopicroside	Glycerin monostearate	OA	Polysorbate80, Poloxamer188	Solvent diffusion	38.19 ± 1.61%	[56]
					129.9 ± 3.07 nm	
Paclitaxel	Cholesterol	OA	Poloxamer188, Polysorbate80	Solvent diffusion	72 ± 11.6%	[57]
Curcumin	CP	Miglyol812	Solutol HS15, Soya lecithin	Film-ultrasonic emulsion evaporation	96.7 ± 0.146%	[58]
					135.3 ± 2.52 nm	
Celecoxib	Kollicream, CP	Miglyol812ic	Solutol HS15, Soya lecithin	low temperature solidification	103.5 ± 32.6 nm	[59]
Amoitone B	Polyethylene glycol stearate GMS	Caprylic/capric triglyceride	Pluronic F68, Soya lecithin	Emulsion evaporation, low temperature solidification	68.17 ± 0.94%	[60]
					225.7 ± 1.36 nm	
Paclitaxel DNA	GMS, Soya lecithin	OA	Polysorbate80	Microemulsion	87.1 ± 2.1%	[61]
					79 nm	
Fenofibrat	Precirol ATO 5	Captex100	Polysorbate80	Melting-emulsification	8.5%	[62]
					227.5 nm	

**Table 2:** Summary of methods for preparing PNPs.

Drugs	Polymer	Surfactant	Surface Modification	Method	Entrapment Efficiency/Particle Size	Ref.
Ketoprofen	EudragitE100 Eudragit RS	---	----	emulsion solvent evaporation	50–150 nm	[64]
TanshinoneIIA	PLGA	Span-80 LABRAFILM 1944 CS, PVA	---	double emulsion evaporation	98.10%	[65]
					188 nm	
Bovine albumin	PLA	PVA	Water soluble chitosan polyethylene	Double emulsion evaporation	100~200 nm	[66]
Paclitaxel	LHRy K	PVA	c(RGDyK)	Dialysis	84.84 ± 2.6%	[67]
					131.7 ± 2.3 nm	
FA	DMAEMA, HEA	---	----	Dialysis	275 nm	[68]
4-Bromo-1,8-naphthalic anhydride	PEI	---	---	Dialysis	5~10 nm	[69]
HCPT	mPEG-LPEI-PCL	PVA	F-CS	Improved thin film dispersion	92.6 ± 1.1%	[70]
					155 ± 9.6 nm	
Paclitaxel	PLGA	Cetyltrimethylammonium bromide	---	Nanoprecipitation	321 ± 0.76 nm	[71]
DNase I	MePEG-PLGA, PEG	---	---	Nanoprecipitation	89.7 nm	[72]
Bovine insulin	PLA	---	---	Supercritical fluid technology	>90%	[73]
					400~600 nm	

**Table 3:** Summary of methods used to prepare PLNs.

Drugs	Lipid	Polymer	Method	Entrapment Efficiency/Particle Size	Ref.
Curcumin	lecithin/cholesterol	PLGA-mPEG, Chol-PEG-RGD	double emulsification	96.0 ± 0.6%	[77]
				216.6 ± 4.7 nm	
HIF1a siRNA	Lecithin, DSPE-PEG-2000, Cholesterol	mPEG-PLGA	double emulsion	141.8 nm	[78]
5-fluorouracil	Phospholipids	mPEG-PLA	improved double emulsion	22.60%	[79]
Oxaplatin	Cholesterol			26.30%	
Camptosar	DSPE-PEG-2000, DSPE-PEG-3400-Mal			96%	
Amoxicillin	Rhamnolipid	PECS	emulsification, solvent evaporation	200 nm	[80]
Human Fibronectin siRNA	DPPC, DSPE-PEG, Phospholipids, DOTAP	curcumin- PLGA	single emulsion, solvent evaporation	~150 nm	[81]
Erlotinib	HSPC, DSPE-PEG-2000	PLGA	DESE	63.3 ± 5.0%	[82]
				213 ± 286 nm	
Doxorubicn	Compritol888, ATO	PCL	single-step sonication	66%	[83]
				170 nm	
Dextran	Soybean lecithin	CT	HPH	~265 nm	[84]
	GTP	HA		27.80%	
	OA			41.50%	
Vancomycin	CHT, ALG	EUD	HPH	54.30%	[85]
Paclitaxel	DSPE-PEG2000	PCL-PEG-PCL	thin-film hydration and ultrasonic dispersion	69.30%	[86]
				279.9 ± 8.7 nm	
Mitomycin C	Phospholipid, Soybean lecithin	PCL	nanoprecipitation	80–90%	[87]
				150–300 nm	
Indocyanine green	DSPE-PEG	PLGA	nanoprecipitation	39 nm, 68 nm	[88]
				116 nm	

## Abbreviations

GTCC	GREOIL GTCC
APG	Alkyl Polyglycoside
ACETEM	Acetylated Mono-and Diglycerides
Lipocire DM	Hydrogenated palm kernel glycerides
Compritol	Compritol 888ATO
mPEG-LPEI-PCL	Methoxy polyethylene-linear polyethyleneimine-poly ε-Caprolactone
DESE	A double emulsion solvent evaporation
